# ISOdb: A Comprehensive Database of Full-Length Isoforms Generated by Iso-Seq

**DOI:** 10.1155/2018/9207637

**Published:** 2018-11-19

**Authors:** Shang-Qian Xie, Yue Han, Xiao-Zhou Chen, Tai-Yu Cao, Kai-Kai Ji, Jie Zhu, Peng Ling, Chuan-Le Xiao

**Affiliations:** ^1^Research Center for Terrestrial Biodiversity of the South China Sea, Institute of Tropical Agriculture and Forestry, Hainan University, Haikou 570228, China; ^2^State Key Laboratory of Ophthalmology, Zhongshan Ophthalmic Center, Sun Yat-sen University, Guangzhou 510060, China; ^3^School of Mathematics and Computer Science, Yunnan Minzu University, Kunming 650031, China

## Abstract

The accurate landscape of transcript isoforms plays an important role in the understanding of gene function and gene regulation. However, building complete transcripts is very challenging for short reads generated using next-generation sequencing. Fortunately, isoform sequencing (Iso-Seq) using single-molecule sequencing technologies, such as PacBio SMRT, provides long reads spanning entire transcript isoforms which do not require assembly. Therefore, we have developed ISOdb, a comprehensive resource database for hosting and carrying out an in-depth analysis of Iso-Seq datasets and visualising the full-length transcript isoforms. The current version of ISOdb has collected 93 publicly available Iso-Seq samples from eight species and presents the samples in two levels: (1) sample level, including metainformation, long read distribution, isoform numbers, and alternative splicing (AS) events of each sample; (2) gene level, including the total isoforms, novel isoform number, novel AS number, and isoform visualisation of each gene. In addition, ISOdb provides a user interface in the website for uploading sample information to facilitate the collection and analysis of researchers' datasets. Currently, ISOdb is the first repository that offers comprehensive resources and convenient public access for hosting, analysing, and visualising Iso-Seq data, which is freely available.

## 1. Introduction

The variability of the transcriptome in an organism accounts for the variations in the phenotype and biological processes [1–4]. The alternative processing of primary RNA transcripts yields diverse spliced forms of the transcripts and mRNA isoforms. These isoforms may differ in structure, function, localization, or other properties [5–7]. Thus, the accurate landscape of transcript isoforms plays an important role in the understanding of gene function and gene regulation. At present, RNA-seq based on next-generation sequencing technology is a widely used approach for transcriptome profiling [8, 9]. While RNA-seq is often challenging to identify full-length gene isoform because of short read assembly, single-molecule real-time sequencing developed by Pacific Biosciences, known as PacBio SMRT, offers an alternative approach to generate longer reads and overcome the disadvantages of RNA-seq. Isoform sequencing (Iso-Seq) developed by PacBio SMRT provides long reads spanning entire transcript isoforms without requirement of assembly [10–12]. Though the PacBio sequencing technology is limited by a lower throughput, higher error rate, and higher cost per base and complemented with RNA-seq to achieve better effects [13], the Iso-Seq still has obvious advantages in improving annotations in reference genomes and identifying gene isoforms, alternative splicing (AS), and gene fusion events. Additionally, it helps in complementing the short/incomplete transcripts for species without a reference genome [14, 15]. However, till date, there is no database that provides comprehensive resources for a complete transcript isoform obtained from Iso-Seq data.

**Figure 1:**
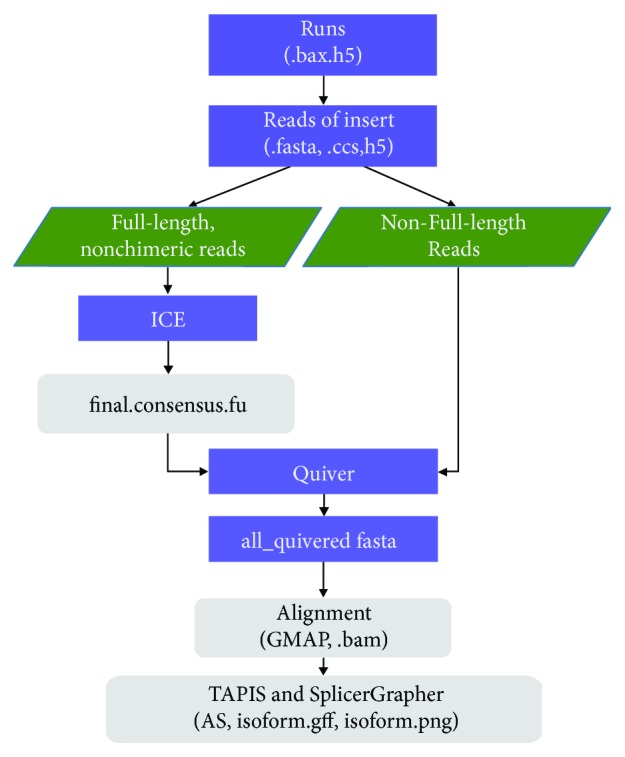
The analysis workflow of Iso-Seq data.

To facilitate the exploration of full-length isoforms in a transcriptome and benefit a broad range of investigators to further understand gene annotations and regulation, we present ISOdb, a comprehensive resource for hosting and carrying out an in-depth analysis of Iso-Seq datasets and visualising the full-length transcript isoforms. The current version of the database has collected 93 publicly available samples from eight species, which were processed and analysed by a unified pipeline (Figure 1). The outputs of ISOdb are presented in two levels: (1) sample level, including metainformation, long read distribution, isoform numbers, and AS events of each sample; (2) gene level, including the total isoform, novel isoform number, novel AS number, and isoform visualisation for each gene. To facilitate further analysis of researcher's datasets and update the database, ISOdb provides a user interface to upload the new sample information and a genome browser to query and visualise the full-length transcript isoforms. ISOdb is publicly available at http://isodb.xieslab.org.

## 2. Methods

### 2.1. Data Collection and Processing

The Iso-Seq data were collected from high-throughput RNA sequence read archive (SRA) database in NCBI. The current version contains 93 samples from eight animals and plants species: *Homo sapiens*, *Mus musculus*, *Gallus gallus*, *Gadus morhua*, *Arabidopsis thaliana*, *Gossypium barbadense*, *Triticum aestivum*, and *Amborella trichopoda*. The analysis tools include SMRT Analysis package, Quiver, GMAP, TAPIS, and SpliceGrapher were used in the pipeline of data processing. The workflow is summarized in Figure 1. Each sample was run through the Iso-Seq pipeline included in the SMRT Analysis software package (https://www.pacb.com/products-and-services/analytical-software/smrt-analysis). First, the raw sequence files produced from PacBio (bax.h5) were extracted, and reads of the insert (known as circular consensus sequence, CCS) were generated using ConsensusTools.sh with the parameters as described in the literature [16]. Subsequently, the reads were classified into full-length and non-full-length reads using pbclassify.py. The full-length reads were fed into the isoform-level clustering (ICE), and all the results were polished using Quiver [17]. Finally, we aligned the quivered fasta sequences against each reference genome by using GMAP [18] and analysed the spliced isoforms with TAPIS and SpliceGrapher by using the annotation file [16].  Table 1 shows the reference genome and related annotation files of the eight species.

### 2.2. Database Implementation

The database was implemented by PHP, MySQL, and JavaScript. The sample and gene information were stored and queried using MySQL and PHP. The JavaScript jQuery and D3.js library were used for producing dynamic and interactive data visualisation in the web browser. In addition, we integrated JBrowse in our database for visualising the full-length isoforms intuitively and the information of alignment against the reference genome for all Iso-Seq sequences in each species, as well as their annotation details were hosted in the genome browser.

## 3. Usage and Features

**Figure 2:**
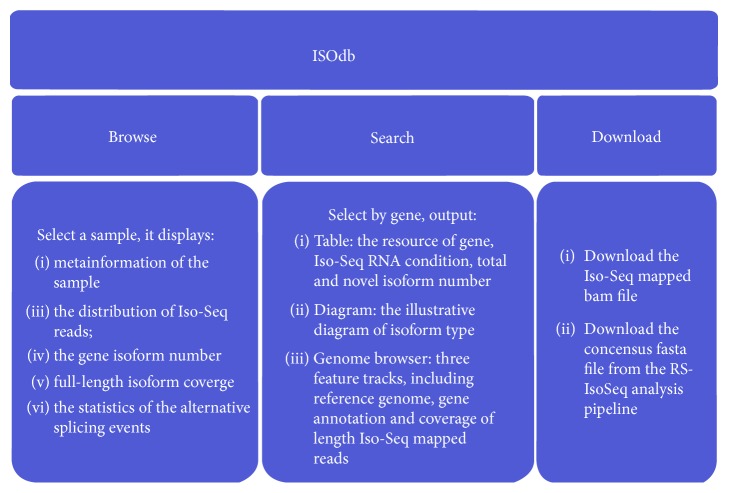
The main functions of ISOdb.

The main function of ISOdb comprises home, browse, search, download, and help pages (Figure 2).

### 3.1. Search

**Figure 3:**
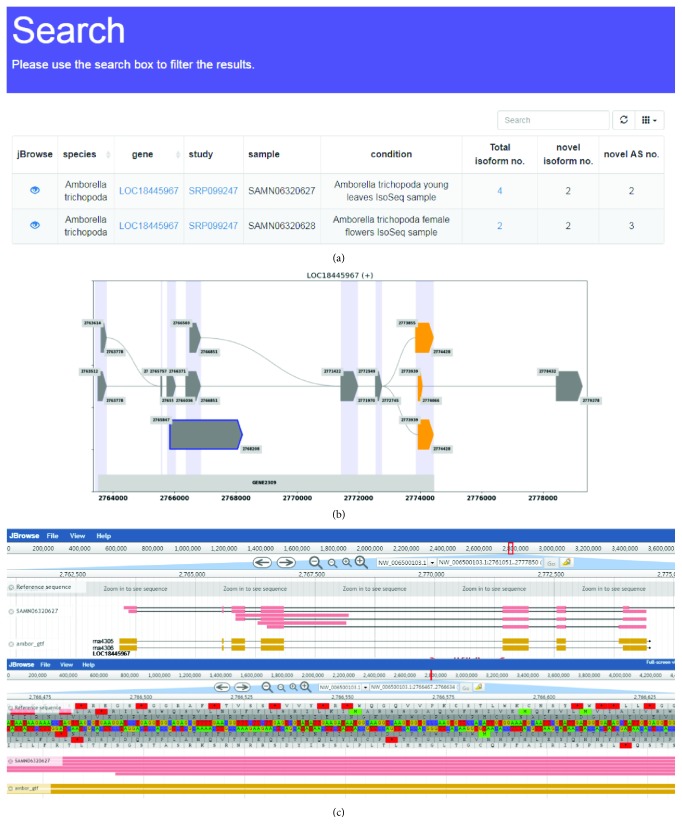
The gene search results from ISOdb. (a) The overview of search output, (b) the detailed diagram of total transcript isoforms (grey block: annotated exon; purple block: alternative 5′ event; orange block: alternative 3′ event; grey block with the blue border: intron retention), and (c) the genome browse of all isoforms.

This page provides a search option for the splice isoforms of genes in the database. Users can search genes by selecting a species and entering a gene symbol or NCBI gene ID in the search box of the search page (also appears in the home page). The output shows the information about the splice isoforms of the gene from all samples for the selected species, including the total isoform number, novel isoform number, and novel AS event number (Figure 3(a) and Figure 3(b)). Based on the transcript annotation file downloaded from NCBI (Table 1), the novel isoforms are identified by TAPIS and diagrammed by SpliceGrapher. In the detailed diagram of transcript isoforms, the grey block is annotated exon, purple block is the alternative 5′ event, orange block is the alternative 3′ event, and grey block with the blue border is intron retention (Figure 3(b)). Besides, the investigators can use a search box on the output page to filter the results. The JBrowse icon provides a hyperlink to a genome browser, which will be described in the next section.

### 3.2. Genome Browser

To explore the distribution of Iso-Seq reads for a given gene, ISOdb provides a genome browser to query and visualise the full-length read coverage and the transcript isoforms. A snapshot of an example of the “genome browser” is shown in Figure 3(c). The annotated gene track and reference are displayed on the top of the browser. Iso-Seq reads of the selected genes are shown at the bottom.

### 3.3. Browse

**Figure 4:**
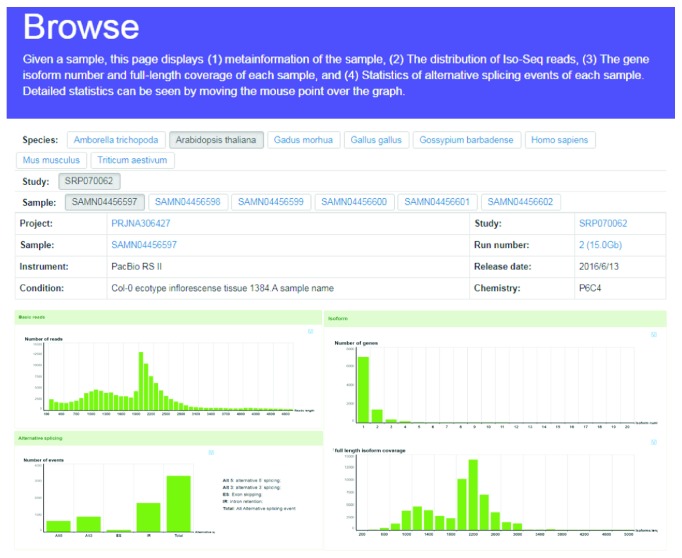
The sample browse results from ISOdb.

For each sample, this page displays (1) metainformation of the sample, including project/study/sample ID, experiment instrument, run number, release date, and experimental condition and PacBio sequencing chemistries (Figure 4); (2) plots showing overall statistics of each sample in three levels: reads, isoforms, and AS events. This section includes read distribution, isoform numbers against gene, full-length isoform numbers, and AS events of each sample (Figure 4).

### 3.4. Download

Investigators can download the bam file of the aligned Iso-Seq reads and consensus fasta sequences of full-length isoforms from each sample. The download page also has the search function so that the users can quickly find out the dataset of their interest for downloading.

## 4. Discussion

ISOdb is the first repository that offers comprehensive resources and convenient public access for hosting, analysing, and visualising Iso-Seq data. The accurate full-length spliced isoforms that are identified by Iso-Seq with no assembly are greatly beneficial for understanding gene annotations and gene regulation. As the numbers of studies using the Iso-Seq technique have been increasing significantly in the recent times, there is a great need for an integrated database that facilitates the exploration of data from Iso-Seq experiments. Thus, we developed the ISOdb by collecting 93 publicly available Iso-Seq samples from eight species and presented the samples along with the metainformation and the full-length splice isoform information of the genes.

Owing to the great advantage of Iso-Seq in identifying full-length transcript isoforms, we envision that Iso-Seq technology will be feasible to apply to a broader set of species and conditions and more such datasets will be released in future. To better collect and analyse datasets from investigators, we provide a user interface in the bottom of the website home page. While more samples are uploaded into ISOdb, some highly sample-dependent isoforms may be obtained. We will make efforts to continue improving the database in a timely manner, and the future updates will include more samples and integrate short reads from RNA-seq to calculate the abundance of transcript isoforms. We hope ISOdb will be a valuable resource for both experimental and computational biologists who are interested in transcriptomics.

## Figures and Tables

**Table 1:** The reference and annotation files for eight species.

Species	Reference	Link
*Amborella trichopoda*	AMTR1.0	https://www.ncbi.nlm.nih.gov/genome/12031
*Arabidopsis thaliana*	TAIR10	http://www.ncbi.nlm.nih.gov/genome/4
*Gadus morhua*	GadMor_May2010	https://www.ncbi.nlm.nih.gov/genome/2661
*Gallus gallus*	Gallus_gallus-5.0	https://www.ncbi.nlm.nih.gov/genome/111 ftp://ftp.ncbi.nih.gov/genomes/Gallus_gallus/GFF
*Gossypium barbadense*	GbV1.0	https://www.ncbi.nlm.nih.gov/genome/10770
*Homo sapiens*	GRCh38.p10	https://www.ncbi.nlm.nih.gov/genome/51
*Mus musculus*	GRCm38.p5	https://www.ncbi.nlm.nih.gov/genome/52
*Triticum aestivum*	CS42_TGAC_v1	http://www.ncbi.nlm.nih.gov/genome/11

## Data Availability

The data used to support the findings of this study are included in the database ISOdb (isodb.xieslab.org).

## References

[1] Lockhart D. J., Winzeler E. A. (2000). Genomics, gene expression and DNA arrays. *Nature*.

[2] Mortazavi A., Williams B. A., McCue K., Schaeffer L., Wold B. (2008). Mapping and quantifying mammalian transcriptomes by RNA-Seq. *Nature Methods*.

[3] Wang B., Tseng E., Regulski M. (2016). Unveiling the complexity of the maize transcriptome by single-molecule long-read sequencing. *Nature Communications*.

[4] Kuang Z., Boeke J. D., Canzar S. (2017). The dynamic landscape of fission yeast meiosis alternative-splice isoforms. *Genome Research*.

[5] Matlin A. J., Clark F., Smith C. W. J. (2005). Understanding alternative splicing: towards a cellular code. *Nature Reviews Molecular Cell Biology*.

[6] Wang E. T., Sandberg R., Luo S. (2008). Alternative isoform regulation in human tissue transcriptomes. *Nature*.

[7] Pan Q., Shai O., Lee L. J., Frey B. J., Blencowe B. J. (2008). Deep surveying of alternative splicing complexity in the human transcriptome by high-throughput sequencing. *Nature Genetics*.

[8] Wang Z., Gerstein M., Snyder M. (2009). RNA-Seq: a revolutionary tool for transcriptomics. *Nature Reviews Genetics*.

[9] Saliba A. E., Westermann A. J., Gorski S. A., Vogel J. (2014). Single-cell RNA-seq: advances and future challenges. *Nucleic Acids Research*.

[10] Sharon D., Tilgner H., Grubert F., Snyder M. (2013). A single-molecule long-read survey of the human transcriptome. *Nature Biotechnology*.

[11] Tseng E., Clark T., Ashby M. H., Shenykman G. (2015). Abstract 4898: full-length isoform sequencing of the human MCF-7 cell line using PacBio long reads. *Cancer Research*.

[12] Shi L., Guo Y., Dong C. (2016). Long-read sequencing and de novo assembly of a Chinese genome. *Nature Communications*.

[13] Rhoads A., Au K. F. (2015). PacBio sequencing and its applications. *Genomics, Proteomics & Bioinformatics*.

[14] Chi K. R. (2016). Finding function in mystery transcripts. *Nature*.

[15] Singh N., Sahu D. K., Chowdhry R. (2016). IsoSeq analysis and functional annotation of the infratentorial ependymoma tumor tissue on PacBio RSII platform. *Meta Gene*.

[16] Abdel-Ghany S. E., Hamilton M., Jacobi J. L. (2016). A survey of the sorghum transcriptome using single-molecule long reads. *Nature Communications*.

[17] Chin C. S., Alexander D. H., Marks P. (2013). Nonhybrid, finished microbial genome assemblies from long-read SMRT sequencing data. *Nature Methods*.

[18] Wu T. D., Watanabe C. K. (2005). GMAP: a genomic mapping and alignment program for mRNA and EST sequences. *Bioinformatics*.

